# Fallskip^®^ Parameters and Their Relationship with the Risk of Falls in Older Individuals with and Without Diabetes

**DOI:** 10.3390/geriatrics10040109

**Published:** 2025-08-08

**Authors:** Azahar Castillo-Montesinos, Lorenzo Brognara, Alejandra Mafla-España, Omar Cauli

**Affiliations:** 1 Department of Nursing, University of Valencia, 46010 Valencia, Spain; azahar.castillo@uv.es (A.C.-M.);; 2 Department of Biomedical and Neuromotor Sciences (DIBINEM), Alma Mater Studiorum University of Bologna, 40123 Bologna, Italy; lorenzo.brognara2@unibo.it; 3 Frailty and Cognitive Impairment Group (FROG), University of Valencia, 46010 Valencia, Spain

**Keywords:** gait, posture, falls, sensors, overweight, diabetes

## Abstract

Background/Objectives: the assessment and prevention of fall risk is an essential component of healthcare, particularly for vulnerable populations such as older adults with or without diabetes. The use of objective and validated tools to assess balance, gait, and other risk factors enables healthcare professionals to make informed clinical decisions and design personalized prevention programs. An observational cross-sectional study was conducted with a probabilistic sample of older patients, with and without diabetes, attending a podiatric clinic (Valencia, Spain). Methods: fall risk was assessed using the Tinetti Scale and the FallSkip^®^ device, which measures posture (i.e., medial-lateral and anterior-posterior displacements), gait (vertical and medial-lateral ranges), turn-to-sit (time) and sit-to-stand (power) tests, total time and gait reaction time. Results: the results showed a significant association between the values obtained with FallSkip^®^ and the Tinetti Scale (*p* < 0.001), identifying the older individuals at high risk of falls. The “reaction time” parameter measured by FallSkip^®^ showed a significant difference between diabetic and non-diabetic patients (*p* < 0.05), as well as the balance score assessed by the Tinetti Scale (*p* < 0.05). Having experienced falls in the previous year had a strong (*p* < 0.001) significant influence on the results evaluated using both the Tinetti Scale and FallSkip^®^. Among the FallSkip^®^ parameters in the multivariate analysis, the ‘Total Time (%)’ parameter significantly (*p* < 0.01, Exp(B) = 0.974 (CI 95%: 0.961–0.988) discriminates individuals with or without falls in the previous year. Conclusions: this study supports the usefulness of the FallSkip^®^ device as an objective, efficient, and easy-to-use tool for fall risk assessment in primary care settings.

## 1. Introduction

Falls are currently the second most common cause of death worldwide among people over 60 years old [1]. One in four older adults falls each year [2]. This is a very heavy financial burden for the healthcare system and for society in general. The most common risk factors are balance disorders, fear of falling, and dementia. There is a higher prevalence among women, and the probability increases with age, as well as among those with a history of previous falls, among other factors [3,4]. An increased risk has also been observed in association with diseases, including Alzheimer’s disease [5,6], Parkinson’s disease [7], and diabetes [8], due to their impact on the nervous system, particularly affecting the patient’s balance and gait [9]. Additionally, people with heart conditions, hypertension, depression, and chronic pain are also at increased risk [10].

Due to prolonged hyperglycemia, patients with diabetes mellitus have a higher likelihood of experiencing falls as a result of muscle weakness, joint stiffness, and comorbidities—especially neuropathy and retinopathy—which affect their balance while walking [10]. The risk of falling in patients with diabetes is 63% higher compared to older adults without diabetes [8].

A wide range of tools to detect fall risk are available, including scales and tests such as the Morse Fall Scale, the St. Thomas’s Risk Assessment Tool [4], the Downton Fall Risk Index [11], the Hendrich II Fall Risk Model [12], the Timed Up and Go Test [13], the Tinetti Scale, and the Berg Balance Scale [14]. All of these are subjective assessment tools sensitive to operator-dependent skills in which the researcher assigns a score to the patient based on the variables defined by each scale. As a result, there is always a slight bias, and they do not demonstrate sufficiently high predictive validity to clearly distinguish between high and low fall risk—specifically, identifying individuals who experience considerable difficulties with mobility, functional abilities and balance [15].

Efforts have been made to develop devices that can objectively measure fall risk, thereby providing more reliable results. One example is the iTUG device, which offers an accessible and cost-effective alternative to enhance the predictive value of the Timed Up and Go test [16]. This innovative tool is designed to be user-friendly, making it accessible even to staff members without specialized training. Unlike conventional sensors, it has a clear interface that simplifies the process of assessing fall risk. This makes it an ideal solution for a podiatry clinic or nursing homes, where timely and accurate evaluations are crucial for ensuring the safety and well-being of residents. By easily integrating this tool into existing routines, practitioners can proactively monitor and respond to potential fall hazards, significantly enhancing the overall care environment.

However, this remains a limited framework, as this method only measures balance. As evidenced by the contributory factors to fall risk, other variables such as posture, gait, and gait speed also play a role [3,13]. Combining at least two assessment tools can therefore lead to a better evaluation of fall-related characteristics in older adults and maximize the advantages of predicting falls in this population [15]. In addition, physicians now have access to different technologies such as inertial sensors [17], video analysis, pressure platforms, and laser sensors, which permit a more comprehensive assessment of fall risk [18]. It is essential that evaluation tools are reliable and comfortable for both the patient and the healthcare professional performing the assessment [19].

FallSkip^®^ is a technological system designed to assess fall risk in older adults and/or individuals with reduced mobility. This device combines inertial sensors with specialized Android-based software to analyze various biomechanical parameters during a series of functional tests. Its goal is to detect early impairments in gait, balance, and mobility, thereby enabling fall prevention through personalized interventions and reducing healthcare costs [18]. It has been used in primary care, rehabilitation, and in the evaluation of post-COVID-19 sequelae due to its speed, simplicity, and ability to provide valuable results, thereby improving the efficiency of care for the elderly population [20,21].

FallSkip^®^ is composed of inertial sensors, including accelerometers and gyroscopes, which are placed on the patient’s lower back, a key area for fall prediction [22]. It includes functional tests such as the Timed Up and Go, static balance, sit-to-stand, and normal and fast-paced walking [23]. The system analyzes parameters including execution time, gait speed, step length, acceleration, postural sway, and symmetry. An integrated analysis software package processes the data and produces an immediate report on the patient’s fall risk [24].

Due to its simplicity and optimization, it has been used in studies with Parkinson’s patients, highlighting impairments in posture, altered gait, and slower sit-to-stand transitions compared to healthy individuals of the same age [23]. Other studies highlight the deterioration based on the stage of the disease in Parkinson’s patients [24,25,26]. Additionally, studies with sarcopenic patients have compared the parameters of the Timed Up and Go (TUG) test with those from the FallSkip^®^ device, with the latter providing more information and greater discriminatory capacity [27,28].

The hypothesis of the study is that the FallSkip^®^ device has been used in few studies to assess fall risk, and more research is therefore needed regarding its use in the diabetic population. Diabetes is an increasingly prevalent health condition that poses a variety of challenges [28], including balance and posture disorders, that can significantly impact patients’ daily lives, heightening their risk of falls—a serious and often debilitating complication [17]. Understanding these issues is crucial to improving the overall well-being and safety of individuals managing diabetes. The primary objective is to evaluate whether there is an association between the parameters provided by the FallSkip^®^ device and the Tinetti Scale. The secondary objective is to determine whether there are significant results for gait and balance impairments in diabetic patients, as assessed by the Tinetti Scale and compared with the results from the FallSkip^®^ device. Third, we wish to analyze which fall-risk tool, i.e., FallSkip^®^ and Tinetti scale, had a more accurate association with having falls or not in the previous year.

## 2. Materials and Methods

### 2.1. Sample Characteristics

This was a cross-sectional observational study, which included a sample of 118 older adults, with and without a diagnosis of type II diabetes mellitus, recruited at the Azahar Podiatric Clinic, located in the province of Valencia, Spain. Participants had to meet the following inclusion criteria: (1) be 60 years of age or older; (2) be able to walk independently or with the aid of a cane or walker; (3) have preserved cognitive function adequate for testing (MMSE ≥ 24). Individuals with the following were excluded: (1) significant hearing impairment; (2) cognitive impairment (MMSE < 24); (3) inability to walk, even with technical assistance. This study was approved by the Ethics Committee of the University of Valencia (Protocol reference: H20190330195344, approval date: 4 April 2019). All participants signed informed consent prior to inclusion in the study. Data collection took place at the Azahar Podiatry Clinic between June 2023 and October 2024.

#### Sample Size

Our study included 118 participants over 60 years of age, with and without a diagnosis of type 2 diabetes mellitus, based on the accessibility and availability of the target population during the data collection period. However, we fully agree on the importance of strengthening methodological rigor through a power analysis, especially with regard to comparisons between subgroups (people with and without diabetes). A power calculation was performed using G*Power software (version 3.1.9.7). An independent samples t-test was used (comparison between groups with and without diabetes), with an estimated effect size of d = 0.5 (medium effect), a significance level of α = 0.05, and the actual group sizes (*n* = X diabetics; *n* = X non-diabetics). The analysis revealed a statistical power (1–β) greater than 80%, with an optimal sample size estimated at 102 participants, confirming that the sample obtained (*n* = 118) was adequate to detect differences with a medium effect between both groups.

### 2.2. Fall Risk Assessment

To carry out this research study, we used the FallSkip^®^ device from Instituto Biomedicina de Valencia IBV (Valencia, Spain), developed by the Valencian Institute of Biomechanics, in order to assess each participant’s fall risk. To that end, four biomechanical variables associated with the likelihood of falling were evaluated: balance, gait, reaction time, sit-to-stand movement, and the total time taken to complete the test. According to the documentation, values closer to 1 indicate a healthier gait pattern [21,29,30].

Based on the calculation of these variables, FallSkip^®^ provides a fall risk score for each patient, ranging from very low, low, moderate, high, to very high risk, depending on the results obtained during the different test phases [30].

The assessment consisted of five phases: the initial balance phase, the approach phase, the sit-to-stand phase, the return phase, and the final balance phase. To begin the test, the device must be placed on the patient using a belt positioned at waist level, aligned with the iliac crest lines.

Initial Balance Phase: The subject must remain standing with their heels slightly apart and their feet forming an angle of approximately 30° to 45°. Their arms should remain relaxed alongside the body. The subject must be told to stay as still as possible, maintaining an upright posture and looking straight ahead. This phase lasts for 30 s.

Approach Phase: Upon hearing the audible signal and reacting as quickly as possible, the patient begins walking toward the chair. Once they reach the chair, they must stop and remain still in that position for about 3 s.

Sit-to-Stand Phase: The patient turns around and sits in the chair without using their arms for support. After remaining seated for 2 s, the patient stands up again without using their arms.

**Figure 1:**
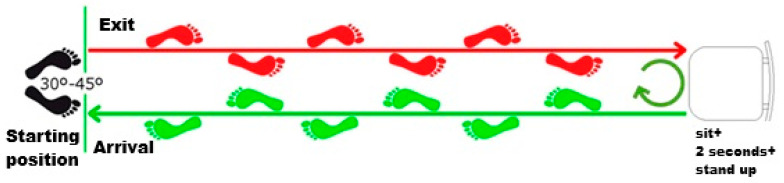
FallSkip^®^ protocol. The patient starts the test when they hear the beep, walks in a straight line to the chair, stops before sitting down, turns around to sit for 2 s, then gets up and continues walking back to the starting point.

Final Balance Phase: The patient remains standing in a balanced position (as in the Initial Balance Phase) until the device indicates the end of the test [4,8]. As shown in Figure 1.

When using the FallSkip^®^ device, the subject’s data is entered (age, weight, height, and whether they have experienced any falls in the previous year). In phase 1 (standing), the subject remains standing and silent for 30 s. Once the device emits a beep, they must begin walking (phase 2). The path is 3 m long and ends at a chair. Once they arrive at the chair, they must stop in front of it, sit down, and then stand up again (phase 3) and start walking back in the opposite direction until they return to the starting point (phase 4). The device will then provide real-time information about the individual’s risk of falling [31].

The FallSkip^®^ has been validated against the standard clinical Timed Up and Go (TUG) test, showing a good correlation (r = 0.70, *p* < 0.001) and greater discriminatory capacity in assessing fall risk in older adults with sarcopenia [27]. Alternatively, a study evaluating an Android app equivalent to FallSkip^®^ reported satisfactory results compared to the Physiological Profile Assessment (PPA) [29]. Regarding reliability, ICCs greater than 0.7 were obtained, as well as adequate values for feasibility and internal consistency in repeated measurements [32,33].

The Tinetti Scale was subsequently administered to each patient. This scale is divided into two parts: balance and gait assessment. The balance evaluation consists of 10 items, which assess sitting balance, standing up, attempts to stand up, immediate balance, standing balance, balance while pushing the patient, balance with eyes closed, 360° turning, and balance when sitting down.

For gait, it assesses the initiation of walking, step length and height (identifying for example if during the step the right foot does not pass the left one), step symmetry, continuity of steps, trajectory (identifying for example whether there is a marked or slight deviation in gait and whether the use of aids is necessary), trunk, and posture while walking. Several items are used to assign the score [34,35,36]. The Tinetti balance is scored out of a maximum of 16 points, and the Tinetti gait is scored out of a maximum of 12 points, with both scores being combined to obtain the final Tinetti scale score, out of a maximum of 28 points. Scores below 19 points are considered a high risk of falling, scores between 19 and 24 points indicate a moderate risk of falling, and 25 points or more indicate a low risk of falling [37,38].

At the same time, we assessed the use of walkers, canes, and free walking, as well as the type of footwear the subject wore, classifying them as slippers with or without insoles, shoes with or without insoles, or Velcro shoes with or without insoles [39,40].

Each patient’s BMI was calculated by dividing their weight in kilograms by their height in square meters, and the result was categorized based on the obtained value. The categories used were underweight (BMI < 18.5), normal weight (BMI 19–24.9), overweight (BMI 25–29.9), and obese (BMI > 30) [41,42,43].

### 2.3. Statistical Analysis

The quantitative variables were reported as mean and standard deviation (SD), and the qualitative variables as frequencies and percentages for descriptive analysis. Additionally, the Kolmogorov test was used to analyze the quantitative variables. The correlations between fall risk variables were analyzed using the Mann−Whitney U test, non-parametric tests, and linear regression multivariate analysis.

All the values obtained were extrapolated to the IBM SPSS statistical software package (version 29.0.2.0), which was used for the entire statistical analysis.

## 3. Results

### 3.1. Characteristics of the Study Sample

The total sample in the study consisted of 118 subjects, of whom 34% were men and 66% were women. The average age of the sample was 76.7 years. Among the participants, 34% were diabetic patients, while 66% belonged to the non-diabetic group. The average BMI of the sample was 28.6. Within the sample, 40% were classified as obese, 38% as overweight, 17% had normal weight, and 4% were underweight. In total, 69 (58.5%) individuals did not use any aid for walking, 21(17.8%) used a walking stick and 28 (23.7%) used a walker. In the study population, 40% experienced a fall during the previous year, compared to 60% who had not fallen within the last year.

### 3.2. Fall Risk Assessed by FallSkip^®^

The fall risk assessed with FallSkip^®^ was low/moderate for 39% of the sample, and 61% presented a high fall risk. Details on the relationship between fall risk and each of the FallSkip^®^ parameters are shown in Table 1.

### 3.3. Fall Risk Evaluated by the Tinetti Scale

The descriptive results obtained from the Tinetti scale were as follows: posture showed a mean score of 11.1 points with a standard error of 3.6, with scores ranging from a minimum of 2 to a maximum of 16. For gait, the mean score was 8.6 points with a standard error of 2.5, with scores ranging from 1 to 15. Finally, the total Tinetti score had a mean of 19.7, with a minimum score of 3 points and a maximum of 28. Scores below 19 points are considered a high risk of falling (N = 46, 39%), scores between 19 and 23 points indicate a moderate risk of falling (N = 35, 29.6%), and 24 points or more indicate a low risk of falling (N = 37, 31.4%). There was a risk of falling (moderate plus high risk pooled together) for 81 individuals (68, 6%).

### 3.4. Association Between FallSkip^®^ and Tinetti Scale to Predict Risk of Falls

In order to analyze the agreement between two ordinal classification tools (Fallskip and Tinetti based on cut-offs), the weighted linear Cohen’s kappa coefficient was applied (Kappa value = −0.025, asymptotic standard error 0.012, Z = −2.487. *p* = 0.013 (lower 95% asymptotic CI bound −0.048 and upper asymptotic CI bound −0.001). Performing correlation analysis between these two ordinal classification tools also led to significant results (tau-b Kendall = −0.336, *p* < 0.001).

When the Tinetti scale score was dichotomized as low versus moderate/high fall risk and the fall risk assessed with FallSkip^®^ was dichotomized as low versus moderate/high fall risk, this lead to a significant relationship between the two tools (*p* ≤ 0.001). Next, we evaluated whether there was a correlation between individual parameters recorded with the FallSkip^®^ (posture, gait, reaction time, sit-to-stand, total time (%), Total time (seconds)) with Tinetti gait, Tinetti balance, and Tinetti total scores.

There is no relationship between the posture variable and the Tinetti parameters (*p* = 0.266, rho = 0.103; *p* = 0.554, rho = 0.055; and *p* = 0.350, rho = 0.087).

**Figure 2:**
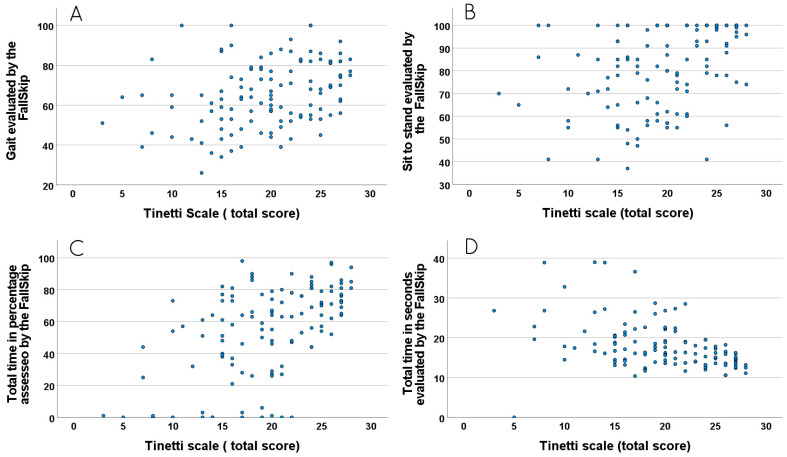
(**A**) Relationship between gait assessed by the FallSkip^®^ and the total score on the Tinetti scale; (**B**) relationship between sit-to-stand assessed by the FallSkip^®^ and the total score on the Tinetti scale; (**C**) relationship between total time in percentage assessed by the FallSkip^®^ and the total score on the Tinetti scale; (**D**) relationship between total time in seconds assessed by FallSkip^®^ and the total score of the Tinetti scale.

However, there is a relationship between the variables gait, sit-to-stand, total time (%) and total time (in seconds), and the Tinetti gait, Tinetti balance, and Tinetti total scores, respectively (*p* < 0.001, rho = 0.470; *p* = 0.12, rho = 0.231; *p* < 0.001, rho = 0.355), (*p* < 0.01, rho = 0.444; *p* < 0.01, rho = 0.341; *p* < 0.01, rho = 0.409), (*p* < 0.01, rho = 0.644; *p* < 0.01, rho = 0.377; *p* < 0.01, rho = 0.516), and (*p* < 0.001, rho = –0.593; *p* < 0.001, rho = –0.330; *p* < 0.001, rho = –0.468), as shown in Figure 2. They all show direct relationships, except for total time in seconds, which presents an inverse relationship with all the Tinetti variables.

### 3.5. Risk of Falls and Falls in the Previous Year

In total, 47 individuals (39.8%) had at least one fall in the previous year. There was no significant effect between having or not having diabetes and having falls or not in the previous year (Chi-squared, *p* = 0.096). The women had more falls than men (higher number of falls in the previous year) (Chi-squared, *p* = 0.006), and those who use a walking stick or walker had more falls compared to those categorized as free walking (*p* < 0.001). Individuals who had falls in the previous year were older (Mann−Whitney test, *p* < 0.001) and had higher BMI (Mann−Whitney test *p* = 0.048).

The chi-square test showed that there is a significant relationship between the fall risk determined by the FallSkip^®^ device and falls in the previous year (*p* < 0.001), as shown in Table 2.

There is no significant relationship between the posture variable provided by the FallSkip^®^ device and having experienced a fall in the previous year (*p* = 0.07). However, there is a significant relationship between having fallen in the previous year and the gait parameter provided by FallSkip^®^ (*p* = 0.01), as well as the reaction time (*p* < 0.01), sit-to-stand (*p* = 0.05), total time percentage (*p* < 0.01), and total time in seconds (*p* < 0.01).

**Figure 3:**
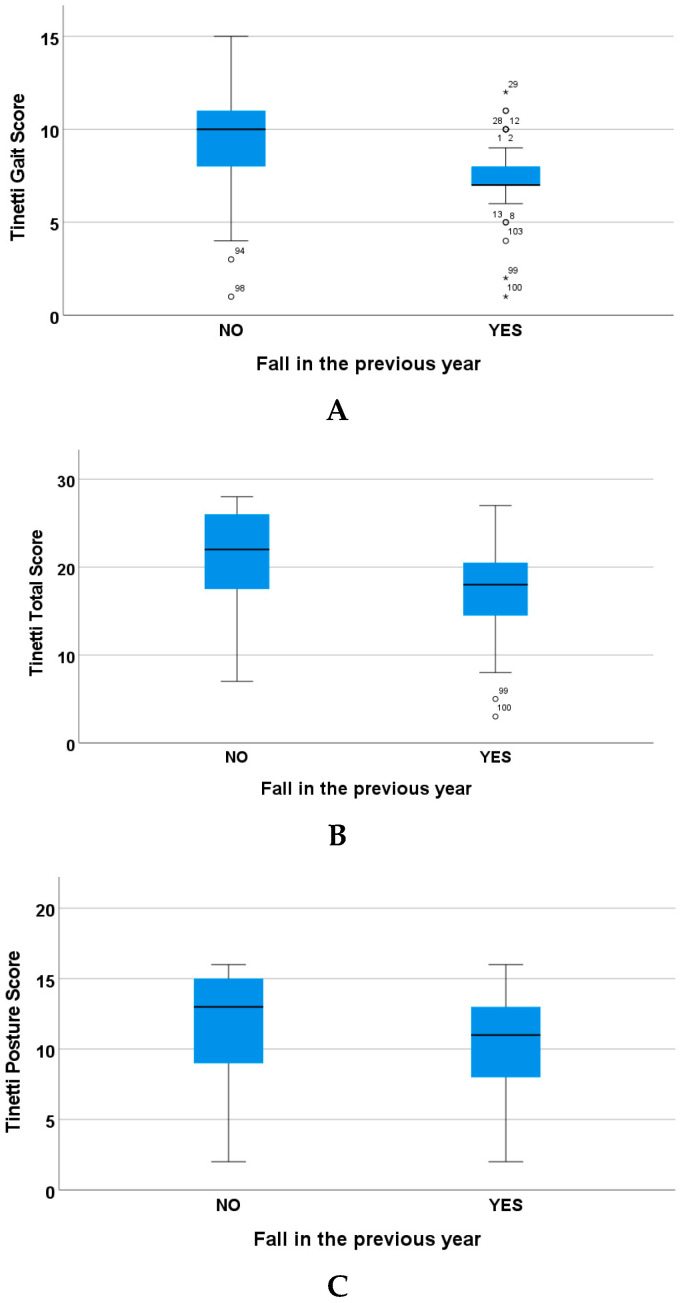
(**A**) Relationship between having experienced a fall or otherwise in the previous year and the Tinetti gait score. (**B**) Relationship between having experienced a fall or otherwise in the previous year and the Tinetti total score. (**C**) Relationship between having experienced a fall or otherwise in the previous year and the Tinetti posture score.

There is a significant difference in the Tinetti parameters between individuals who have experienced falls in the previous year and those who have not (Tinetti gait *p* < 0.001, Tinetti posture *p* = 0.017, Tinetti total score *p* < 0.001), as shown in Figure 3.

### 3.6. Risk of Falls in Diabetic and Non-Diabetic Patients

**Figure 4:**
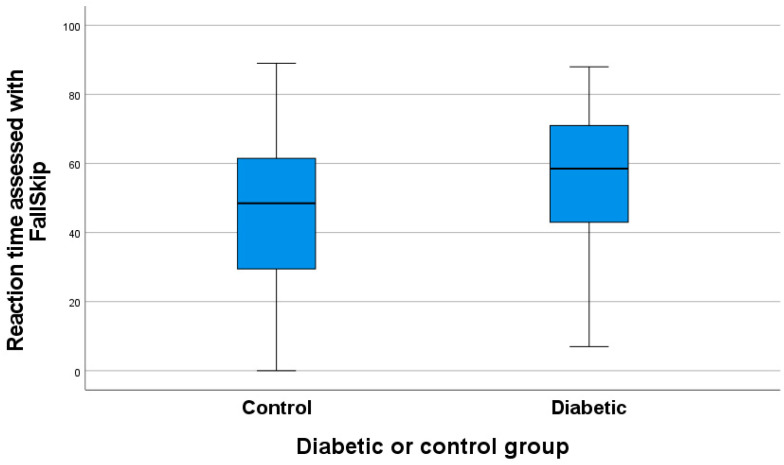
Reaction time assessed with FallSkip^®^ in diabetic patients and the non-diabetic group.

There is no significant difference in the posture parameter between individuals with diabetes and those without (*p* = 0.62). Similarly, there is no difference for the gait variable (*p* = 0.99), the sit-to-stand variable (*p* = 0.82), or the total time variable (*p* = 0.11). However, there is a trend toward a difference in the reaction time variable between diabetic and non-diabetic participants (*p* = 0.048), as shown in Figure 4. The mean reaction time in non-diabetic individuals was 45.96 ± 2.62, compared to 54.58 ± 3.38 in diabetic individuals 

**Figure 5:**
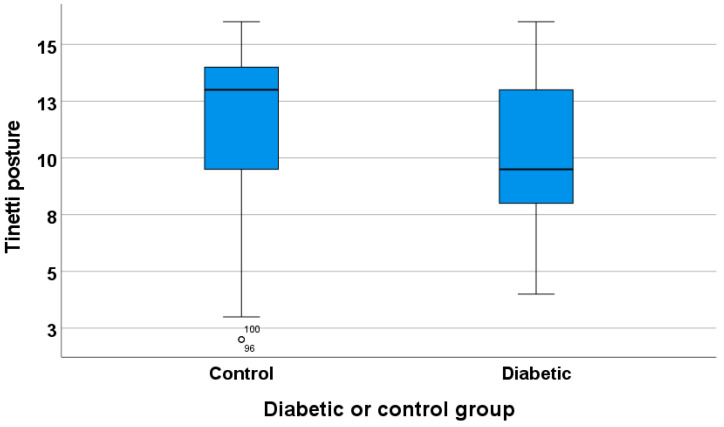
Tinetti posture in the diabetic and non-diabetic group.

There is no significant difference between diabetic patients and non-diabetic patients in the parameters assessed with the Tinetti gait score (*p* = 0.830) and the total Tinetti score (*p* = 0.135). However, there is a significant difference between diabetic patients and non-diabetic patients in the Tinetti posture parameters (*p* = 0.049), as shown in Figure 5.

### 3.7. Gait and Posture Parameters Measured FallSkip^®^ and Tinetti Scale as Predictors of Falls in the Previous Year

Multivariate analysis was performed in order to analyze which variable predicts falls in the previous year (dependent variable). Independent variables included age, sex, walking aid, BMI, and each FallSkip^®^ parameter (posture, gait, reaction time, sit to stand, total time (%), total time (seconds)) with Tinetti gait and Tinetti balance score. Logistic regression analysis showed that falls in the previous year were significantly associated with gender (female *p* = 0.031) and the use of an aid for walking (*p* = 0.001 for walking stick and *p* = 0.007 for walker) (Cox and Snell R-squared 0.340, Nagelkerke R-squared 0.460, Hosmer−Lemeshow, chi-squared 1.234, significant 0.996). These results are shown in the Table 3.

## 4. Discussion

Assessing fall risk in the adult population is essential, as it facilitates the prevention of injuries, enhances quality of life, and contributes to reducing healthcare expenditure, given that over 25% of adults experience at least one fall per year [2]. Early identification of individual risk factors enables the implementation of targeted interventions to mitigate the likelihood of falls [2,44,45]

Primary care physicians can identify and optimize risk factors to reduce the likelihood of falls [43]. They have access to a variety of validated assessment tools for evaluating patients. However, many of these tools are time-consuming and involve a significant degree of subjectivity [14,15]. In 2007, Scott et al. [44] concluded that none of the 38 available fall risk assessment tests could be recommended for use in isolation. The evidence also suggests that combining exercise programs, medication reviews, patient education, continuous assessment, staff training, and the integration of technological advancements can effectively reduce the incidence of falls and enhance patient safety in healthcare settings [46,47]. In the present study, we demonstrate the FallSkip^®^ device, which is still relatively novel and for which limited research is available [26,27] but is a reliable tool for identifying individuals among the older population at risk of falling. It also permits the analysis of additional variables influencing fall risk, such as the total time required for a patient to complete the test. FallSkip^®^ enables objective assessment free from investigator bias and offers high levels of specificity in determining a person’s level of fall risk [30,34]. Our findings show that while the average score on the Tinetti scale suggests a moderate risk, FallSkip^®^ offers greater precision by classifying the population as having a high fall risk, specifically on recognizing those individuals who face significant challenges with mobility, functional abilities, and balance. Their experiences are unique and deserve our attention and understanding.

The Tinetti scale has been widely used in numerous studies to assess fall risk in adults. Jahantabi-Nejad et al. [46] recommend the use of this scale, particularly when a more detailed analysis of balance is needed, such as single-limb static stability or anticipatory postural adjustments. However, other authors such as Lusardi et al. [47] and Hars et al. [48] do not recommend relying on the Tinetti scale as the sole tool for predicting fall risk in adults. For this reason, we compared the results obtained using the Tinetti scale and the FallSkip^®^ device in our sample to ascertain whether there were biases between a subjectively obtained score and one derived objectively, using a scale backed by years of scientific validation and a relatively novel technological tool. Based on this comparison, we found a correlation between gait-related variables, but not in the assessment of balance, where evaluator bias may have influenced the results. This bias highlights the discrepancy in posture-related measurements obtained using the Tinetti scale compared to those generated by the FallSkip^®^ device in our study. The evaluation method, objectivity, and data accuracy are influential factors. In the Tinetti scale, the assessment relies solely on the clinician and their clinical judgment, whereas the FallSkip^®^ device utilizes inertial sensors (accelerometers and gyroscopes) that objectively record the results. The Tinetti scale assesses posture through general observations (e.g., whether the patient sways or maintains balance), without providing detailed quantitative measurements. In contrast, FallSkip^®^ can detect postural micro-oscillations, reaction times, displacements of the center of mass, and other precise kinematic parameters related to postural stability, even in the early stages of deterioration. Therefore, it can be stated that the Tinetti scale evaluates posture in a more qualitative and subjective manner, whereas FallSkip^®^ offers a quantitative, objective, and accurate assessment.

Polyneuropathy is one of the main complications of diabetes and leads to a loss of postural stability, making it a potential risk factor for falls. Estimates suggest that approximately 40% of falls among older adults occur in diabetic patients, with the risk being 63% higher in individuals with type II diabetes compared to non-diabetic adults [8]. When evaluating FallSkip^®^ results in both diabetic and non-diabetic individuals, we observed a significant difference only in the reaction time, with diabetic patients showing longer reaction times. This finding aligns with the study by Assar et al. [49], which demonstrated that diabetic patients exhibited delayed responses to postural changes and a loss of muscle strength due to muscular atrophy, which is a consequence of diabetes-related polyneuropathy.

Patients suffering from diabetic foot conditions often do not receive thorough assessments of their fall risk during visits to podiatry clinics. This study shows that recent technological breakthroughs have introduced innovative tools that are accessible even to non-expert practitioners. These tools facilitate a more comprehensive screening process, enabling the early detection of complications that could lead to severe health issues. By integrating these technologies into routine care, podiatrists can improve patient outcomes and enhance safety for individuals at risk.

As a limitation of the study, we did not have access to each patient’s glycated hemoglobin (HbA1c) value, which introduced a bias in our study [50,51]. Conducting a study in which the HbA1c value of each patient is known would therefore be worthwhile in order to obtain more accurate information about glycemic control of individuals with diabetes. This is particularly relevant given that blood glucose variability has been identified as a potential risk factor for falls [52,53].

There has been an increase in cases of neuropathy, retinopathy, and amputations among patients with lower socioeconomic status [54]. This lack of adherence to care can lead to the development of ulcers, which have been shown to be a predisposing factor for falls due to the imbalance they cause during gait. Additionally, the patient’s level of education has also been found to influence this risk [8].

In our study, in multivariate analysis, we found that the strong predictors of falls in the previous year were age, sex, and the use of a walker, and these are three factors that significantly influence the likelihood of experiencing a fall [9,55]. Advanced age is associated with a decline in physical condition, affecting balance, posture, and the auditory system, which consequently increases the risk of falling [2]. A higher fall risk has been observed in females [56]. Gazibara et al. [55] reported that being female and having a fear of falling are strong predictors of falls in older adults. Furthermore, Stevens et al. confirmed that women are more likely to report falls, seek medical attention, and engage in conversations about fall prevention [56].

In this case, BMI was not found to be significant for the study, but it does influence the fall risk. This is because BMI calculation does not account for a person’s muscle mass. Previous reports by Colón-Emeric et al. [2], Bayrak et al. [11], and Assar et al. [49] pointed out that muscle mass loss affects balance and consequently increases the fall risk in adults. For this reason, fall risk prevention plans include exercise programs aimed at strengthening muscles [44]. Cho et al. [57] confirmed in their study that obese patients have a higher likelihood of falling and experience more frequent falls than individuals with normal weight, but obese individuals who do fall tend to experience fewer injuries than those with normal weight. Future studies taking into account lean and fat mass besides BMI are warranted in order to provide new insights into fall risk assessed with this device and body mass composition.

### 4.1. Study Limitations

This study has several limitations that should be taken into account when interpreting the results. First, none of the participants with type 2 diabetes had serious foot complications, such as active ulcers or amputations. Therefore, even more pronounced differences in the parameters measured by the FallSkip^®^ device cannot be ruled out if a population with advanced complications had been included. The exclusion of these cases limits the generalizability of the findings to patients with more complex diabetes or in more advanced stages of the disease.

Second, data were not collected on the participants’ socioeconomic status, a variable that has been shown to be closely related to the prevalence of diabetic complications such as neuropathy, retinopathy, and amputations. Several studies indicate that patients with diabetes of lower socioeconomic status have a higher burden of complications due to, among other factors, less access to health services and poor therapeutic adherence [49]. This lack of adequate care can lead to the development of foot ulcers, which have been identified as a predisposing factor for falls, significantly altering stability and gait patterns. Furthermore, educational level also influences the risk of falls, possibly mediated by differences in knowledge about the disease, adherence to clinical recommendations, and the ability to make self-care decisions [8].

The presence of peripheral neuropathy and diabetic retinopathy was also not specifically assessed, despite both being widely documented as key risk factors for increased falls in older adults with diabetes [58,59,60,61]. The absence of these clinical variables limits the study’s ability to fully explain the differences observed between the subgroups with and without diabetes and should therefore be considered a significant limitation of the design.

On the other hand, although glycated hemoglobin (HbA1c) levels were not measured in our sample, scientific evidence shows that both the excessively strict glycemic non-diabetic group (HbA1c ≤ 6–7%) and poor non-diabetic group (HbA1c ≥ 8%) are associated with an increased risk of falls, especially in older patients treated with insulin. In this context, intermediate HbA1c levels (~7%) appear to offer a better balance between preventing hypoglycemia and protecting against fractures or falls [62]. Therefore, the lack of data on HbA1c represents a significant limitation, as it could have influenced the functional differences observed in the sample.

It should be noted that, as a non-diabetic group measure, only individuals with preserved cognitive function (MMSE ≥ 24) and the ability to walk, either independently or with the aid of devices such as a cane or walker, were included. This selection partially contributed to reducing the bias associated with severely impaired physical and cognitive functioning.

### 4.2. Strengths of the Study

One of the main strengths of this study is the demonstration of a significant association between parameters provided by the FallSkip^®^ device and the Tinetti scale, which supports the concurrent validity of the former as a tool for assessing fall risk in older adults. Both instruments demonstrated consistency in identifying high-risk individuals, reinforcing the clinical utility of FallSkip^®^ as an objective, rapid, and technologically advanced alternative. Furthermore, the significant relationship between FallSkip^®^ test results and a history of falls in the past year reinforces its predictive validity, although longitudinal studies are necessary to confirm this. This indicates that the device not only measures functional parameters but also has direct clinical relevance. The study’s implementation in a healthcare setting, specifically in a podiatry clinic, confers high validity to the study, as it reflects routine healthcare conditions and allows for the assessment of the practical applicability of the FallSkip^®^ device in everyday clinical settings. Finally, the inclusion of a heterogeneous sample of older adults with and without type 2 diabetes allows for greater generalization of the findings and opens the door to exploring relevant clinical differences between subgroups.

### 4.3. Future Lines of Research

Based on the results obtained and the limitations identified in this study, several future lines of research are proposed. First, it would be relevant to incorporate additional clinical variables in people with type 2 diabetes, such as glycated hemoglobin (HbA1c) levels, disease duration, and the presence of chronic complications such as peripheral neuropathy or retinopathy, given their potential influence on the risk of falls. Furthermore, future studies could analyze the impact of socioeconomic and educational status on diabetes control and its relationship with physical function and balance.

We also suggest conducting longitudinal studies to evaluate the predictive capacity of the FallSkip^®^ device in medium- and long-term follow-up, as well as its usefulness in detecting changes in the risk of falls after specific interventions (e.g., exercise programs, therapeutic education, or improved glycemic control). On the other hand, it would be interesting to explore the device’s consistency with other objective or clinical tools in different healthcare settings (such as nursing homes, primary care, or hospital services), which would allow for establishing applicability in broader contexts. Finally, future research could expand the sample to include people with higher levels of functional dependency in order to assess the device’s usefulness in more vulnerable populations.

## 5. Conclusions

This study has demonstrated a significant association between the parameters provided by the FallSkip^®^ device and the Tinetti scale in assessing the risk of falls in older adults. Statistical analysis has shown that both tools are consistent in identifying patients at high risk of falling. The significant relationship between the fall risk assessed by FallSkip^®^ and the incidence of falls in the past year reinforces the validity of this device as a tool for evaluating fall risk. Furthermore, parameters such as “Gait,” “Sit to Stand,” “Total Time %” and “Total Time in Seconds” were found to be related to variables on the Tinetti scale, indicating that FallSkip^®^ can effectively complement traditional clinical assessments. Additionally, it was observed that the “reaction time” variable measured by FallSkip^®^ shows a significant difference between diabetic and non-diabetic patients and is longer for the former group. Similarly, the balance subscore of the Tinetti scale shows a significant difference between the two groups, suggesting that diabetes may influence balance and gait impairments.

Our findings show that sex and age are factors that influence the likelihood of falling among this population. In conclusion, the results support the usefulness of the FallSkip^®^ device as an objective and efficient tool for assessing fall risks. Its implementation in clinical settings could contribute to a more accurate identification of at-risk patients and the personalization of preventive strategies. Longitudinal analyses are necessary in order to use the Fallskip device as a sensitive measurement to detect gait and posture alterations in older individuals, irrespective of the presence of diabetes.

## Figures and Tables

**Table 1:** Relationship between risk of falls measured by FallSkip^®^ (dichotomized in low versus moderate/high fall risk) with each FallSkip^®^ parameter.

	Low Fall Risk	Moderate−High Fall Risk	*p* Value	Effect Size Cohen’s D; Effect-Size R
Posture	Mean ± SD: 78.1 ± 20.1 (Minimum 0–Maximum 99)	Mean ± SD: 77.1 ± 16.0 (Minimum 14–Maximum 100)	*p* = 0.402	0.055; 0.027
Gait	Mean ± SD: 72.9 ± 13.3 (Minimum 45–Maximum 100)	Mean ± SD: 62.1 ± 16.5 (Minimum 26–Maximum 100)	*p* ≤ 0.001	0.667; 0.316
Reaction time	Mean ± SD: 59.2 ± 19.9 (Minimum 1–Maximum 89)	Mean ± SD: 43.9 ± 22.8 (Minimum 0–Maximum 88)	*p* ≤ 0.001	0.715; 0.337
Sit to stand	Mean ± SD: 91.8 ± 13.5 (Minimum 41–Maximum 100)	Mean ± SD: 76.2 ± 17.7 (Minimum 0–Maximum 88)	*p* ≤ 0.001	0.991; 0.444
Total time %	Mean ± SD: 73.4 ± 13.2 (Minimum 44–Maximum 97)	Mean ± SD: 46.8 ± 28.8 (Minimum 0–Maximum 98)	*p* ≤ 0.001	0.918; 0.417
Total time seconds	Mean ± SD: 14.5 ± 2.3 (Minimum 10.6–Maximum 19.5)	Mean ± SD: 19.4 ± 6.7 (Minimum 0–Maximum 39)	*p* ≤ 0.001	−0.978; −0.439

**Table 2:** Relationship between falls in the previous year and FallSkip^®^ parameters.

	No Falls in the Last Year	Falls in the Last Year	*p* Value	Effect Size Cohen’s D; Effect-Size R
Posture	Mean ± SD: 79.3 ± 17.2 (Minimum 0–Maximum 100)	Mean ± SD: 74.6 ± 17.2 (Minimum 14- Maximum 100)	*p* = 0.79	0.273; 0.135
Gait	Mean ± SD: 69.3 ± 15.4 (Minimum 34–Maximum 100)	Mean ± SD: 59.7 ± 15.9 (Minimum 26- Maximum 100)	*p* = 0.01	0.613; 0.293
Reaction time	Mean ± SD: 54.7 ± 21.5 (Minimum 0–Maximum 89)	Mean ± SD: 39.6 ± 22.2 (Minimum 0- Maximum 88)	*p* ≤ 0.01	0.691; 0.326
Sit to stand	Mean ± SD: 85.2 ± 16.5 (Minimum 37–Maximum 100)	Mean ± SD: 74.9 ± 18.5 (Minimum 41–Maximum 100)	*p* = 0.005	0.588; 0.282
Total time (%)	Mean ± SD: 63.7 ± 24.1 (Minimum 0–Maximum 97)	Mean ± SD: 42.1 ± 28.1 (Minimum 0–Maximum 98)	*p* ≤ 0.01	0.825; 0.381
Total time (seconds)	Mean ± SD: 16.4 ± 5.0 (Minimum 10.6–Maximum 38.9)	Mean ± SD: 20.0 ± 7.0 (Minimum 0–Maximum 39)	*p* ≤ 0.01	−0.592; −0.284

**Table 3:** Logistic regression analysis to assess which variables were associated with having or not having falls in the previous year.

	Beta (B) Coefficients	Standard Error	Wald	Gl	*p* Value	Exp (B)	95% C.I. for EXP (B)	
							Lower	Upper
Posture	–0.023	0.014	2.429	1	0.119	0.978	0.950	1.006
Gait	–0.012	0.020	0.370	1	0.543	0.988	0.951	1.027
Reaction time	0.003	0.015	0.038	1	0.845	1.003	0.975	1.032
Sit to stand	–0.012	0.016	0.576	1	0.448	0.988	0.957	1.020
Total time %	0.000	0.026	0.000	1	0.997	1.000	0.950	1.053
Total time (seconds)	–0.082	0.100	0.670	1	0.413	0.921	0.757	1.121
Tinetti gait	–0.001	0.175	0.000	1	0.996	0.999	0.709	1.408
Tinetti balance	–0.066	0.097	0.464	1	0.496	0.936	0.775	1.132
BMI			1.535	3	0.674			
BMI (1)	–0.912	1.460	0.390	1	0.532	0.402	0.023	7.024
BMI (2)	–0.721	0.744	0.940	1	0.332	0.486	0.113	2.090
BMI (3)	0.114	0.576	0.039	1	0.843	1.121	0.362	3.467
Gender (women versus men)	1.286	0.596	4.664	1	0.031	3.620	1.126	11.632
Use of aid for walking			10.993	2	0.004			
Walking stick (1)	2.965	0.924	10.301	1	0.001	19.397	3.172	118.608
Walker (2)	2.654	0.984	7.268	1	0.007	14.210	2.064	97.846
Diabetics versus non diabetic l (1)	–0.346	0.598	0.335	1	0.563	0.707	0.219	2.285
Constant	3.218	3.805	0.715	1	0.398	24.971		

## Data Availability

Research data will be shared on reasonable request to the corresponding author.
